# A rapid screening method for the detection of specialised metabolites from bacteria: Induction and suppression of metabolites from *Burkholderia* species

**DOI:** 10.1016/j.mimet.2020.106057

**Published:** 2020-11

**Authors:** Gordon Webster, Cerith Jones, Alex J. Mullins, Eshwar Mahenthiralingam

**Affiliations:** Microbiomes, Microbes and Informatics Group, Organisms and Environment Division, School of Biosciences, Cardiff University, The Sir Martin Evans Building, Museum Avenue, Cardiff, Wales CF10 3AX, UK.

**Keywords:** Bacteria, Specialised metabolites, Antibiotics, *Burkholderia*, Antibiotic discovery, HPLC

## Abstract

Screening microbial cultures for specialised metabolites is essential for the discovery of new biologically active compounds. A novel, cost-effective and rapid screening method is described for extracting specialised metabolites from bacteria grown on agar plates, coupled with HPLC for basic identification of known and potentially novel metabolites. The method allows the screening of culture collections to identify optimal production strains and metabolite induction conditions. The protocol was optimised on two *Burkholderia* species known to produce the antibiotics, enacyloxin IIa (*B. ambifaria*) and gladiolin (*B. gladioli*), respectively; it was then applied to strains of each species to identify high antibiotic producers. *B. ambifaria* AMMD and *B. gladioli* BCC0238 produced the highest concentrations of the respective antibiotic under the conditions tested. To induce expression of silent biosynthetic gene clusters, the addition of low concentrations of antibiotics to growth media was evaluated as known elicitors of *Burkholderia* specialised metabolites. Subinhibitory concentrations of trimethoprim and other clinically therapeutic antibiotics were evaluated and screened against a panel of *B. gladioli* and *B. ambifaria*. To enhance rapid strain screening with more antibiotic elicitors, antimicrobial susceptibility testing discs were included within the induction medium. Low concentrations of trimethoprim suppressed the production of specialised metabolites in *B. gladioli*, including the toxins, toxoflavin and bongkrekic acid. However, the addition of trimethoprim significantly improved enacylocin IIa concentrations in *B. ambifaria* AMMD. Rifampicin and ceftazidime significantly improved the yield of gladiolin and caryoynencin by *B. gladioli* BCC0238, respectively, and cepacin increased 2-fold with tobramycin in *B. ambifaria* BCC0191. Potentially novel metabolites were also induced by subinhibitory concentrations of tobramycin and chloramphenicol in *B. ambifaria*. In contrast to previous findings that low concentrations of antibiotic elicit *Burkholderia* metabolite production, we found they acted as both inducers or suppressors dependent on the metabolite and the strains producing them. In conclusion, the screening protocol enabled rapid characterization of *Burkholderia* metabolites, the identification of suitable producer strains, potentially novel natural products and an understanding of metabolite regulation in the presence of inducing or suppressing conditions.

## Highlights

• A rapid screening method for extracting specialised metabolites from *Burkholderia* and other bacteria was developed
• The method allowed screening of culture collections to identify production strains and metabolite induction conditions
• Screening provided an understanding of metabolite regulation in the presence of different inducing and suppressing conditions

## Graphical abstract

**Figure:**
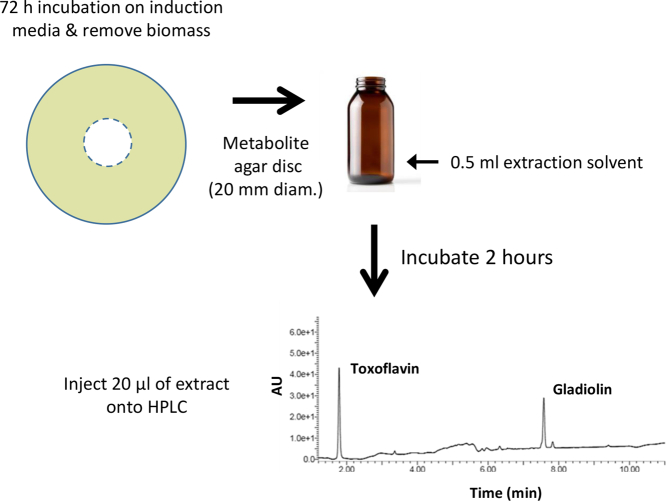

## 1 Introduction

Microbial specialised (also known as secondary) metabolites continue to be a source of new biologically active molecules for use in medicine and agriculture (Bérdy 2005; Rutledge and Challis 2015). However, their production, extraction and identification from bacterial growth medium can be complicated, labour intensive, and time consuming. Natural product extraction methods from microbial sources frequently involve the use of freeze-drying, evaporation under vacuum, adsorption to ion-exchange resins, or use of large volumes of harmful solvents (Seidel 2012; Sterner 2012). In addition, the production of microbial compounds are frequently influenced by different cultivation parameters (e.g. nutrients, light, temperature, pH, and aeration) (Bode et al. 2002, Pettit 2011, Begani et al. 2018) and identifying optimum growth conditions can require labour intensive screening. This has prompted the investigation of alternative approaches to identifying novel metabolites, such as high throughput screening (HTS) of synthetic compound libraries and fragment-based design (Payne et al. 2006; Doak et al. 2016). However, these approaches have had limited success due to the nature of the developed assay to screen large numbers of compounds. In both cases, inhibition of the target protein by the tested compounds is assessed outside the context of the cell, and unfortunately lead compounds are often found to be ineffective in cell-based assays (Rutledge and Challis 2015).

The recent explosion in microbial genome sequencing projects (Mukherjee et al. 2017) and ever-increasing computational capacity has allowed for the powerful approach of genome mining to be realised. Genome sequencing coupled with specific genome mining tools, such as antiSMASH (Medema et al. 2011), has revealed that multiple microorganisms, beyond the traditionally exploited *Streptomyces* genus (Hopwood 2019) have excellent potential to produce specialised metabolites encoded by biosynthetic gene clusters (BGCs) (Trivella and de Felicio 2018). Recently, antibiotics and other bioactive molecules have been identified in members of the *Betaprotebacteria* genus *Burkholderia* (e.g. Mahenthiralingam et al. 2011; Ross et al. 2014; Song et al. 2017; Flórez et al. 2018; Jenner et al. 2019; Mullins et al. 2019; Jones et al. 2020). Novel gene clusters identified by genome mining also demonstrate that *Burkholderia* carry multiple silent or cryptic biosynthetic loci with untapped metabolite potential (Depoorter et al. 2016; Kunakom and Eustáquio 2019; Mullins et al. 2020). However, despite the identification of novel BGCs, activating these silent gene clusters continues to present a major challenge.

The addition of induction molecules or chemical elicitors to growth media, such as glycerol has been used routinely for metabolite investigations with *Burkholderia* species (Keum et al. 2009; Mahenthiralingam et al. 2011; Song et al. 2017; Mullins et al. 2019). Subinhibitory concentrations of clinically used antibiotics (e.g. trimethoprim and piperacillin); (Seyedsayamdost 2014), have also been shown to induce specialised metabolites in *Burkholderia thailandensis*. Systematic investigation into elicitors that awaken silent BGCs would have a major impact on drug discovery (Begani et al. 2018). Therefore, a rapid screening method to aid compound identification and obtain optimal producer strains, as well as allow the user to determine conditions needed to express these novel compounds, is urgently required to unlock the genetic potential of *Burkholderia* and other antibiotic producing microorganisms.

Here we describe a novel, highly efficient screening method based on solvent extraction of specialised metabolites directly from agar plate cultures, coupled with reversed-phase high-performance liquid chromatography (HPLC) as a basic and widely available compound profiling analysis. We expanded the protocol to incorporate antibiotic susceptibility testing discs in the agar, and rapidly screen large panels of *Burkholderia* strains for novel metabolite induction or suppression properties caused by these gene expression altering antimicrobials. The method allowed the identification of known and novel compounds, screening novel chemical elicitors, and identification of optimal production strains and growth/metabolite induction conditions. In this study we evaluated *Burkholderia* as specialised metabolite producers, but the method could readily be employed for other bacteria which demonstrate similar growth properties to these rapidly growing Gram-negative bacteria.

## 2 Materials and methods

### 2.1 Bacterial strains and growth conditions

All strains of *Burkholderia* (Table 1) were drawn from the Cardiff University *Burkholderia* culture collection (Mahenthiralingam et al. 2011; Mullins et al. 2020) and other recognised strain repositories (The Belgium Co-ordinated Collections of Microorganisms/Laboratory of Microbiology, Ghent [BCCM/LMG]; The *Burkholderia cepacia* Research Laboratory and Repository [BcRLR]), and stored at −80 °C in Tryptone Soya Broth (TSB; Oxoid) containing 8% (v/v) dimethylsulfoxide (DMSO; Sigma). Cultures were revived onto Tryptone Soya Agar (TSA; Oxoid) in Petri dishes and incubated at 30 °C for 24 h. All cultures were routinely streaked to single colonies on TSA to check for purity. Overnight liquid cultures were prepared by inoculating 5 ml of TSB with confluent growth from a fresh TSA plate, incubated at 30 °C on a rocking platform (150 rpm) and used as bacterial inoculum of agar medium for specialised metabolite induction.

**Table 1:** *Burkholderia* species strains used in this study.

Strain name	Alternative strain name(s)	Source details	Specialised metabolites known to be produced	References
*Burkholderia ambifaria*				
AMMD	LMG 19182^T^, ATCC BAA-244^T^	Pea rhizosphere, USA	Enacyloxin, pyrrolnitrin, burkholdines, AFC-BC11, hydroxyquinolines	Coenye et al. (2001); Mahenthiralingam et al. (2011); Mullins et al. (2019)
BCC0118	CEP0617, R-9917	CF patient sputum, USA	Enacyloxin, pyrrolnitrin, burkholdines, AFC-BC11, hydroxyquinolines	Coenye et al. (2001);
BCC0191	Bc-B, ATCC 51993, J82, R-5140	Soil, USA (biocontrol strain)	Cepacin, pyrrolnitrin burkholdines, phenazine	Mao et al. (1997); Mullins et al. (2019)
BCC0203	Bc-F, HG1-A	Maize rhizosphere, USA (biocontrol strain)	Enacyloxin, pyrrolnitrin, burkholdines, bactobolins, AFC-BC11	Mao et al. (1998); Mullins et al. (2019)
BCC0207	AMMD^T^, LMG 19182^T^	AMMD^T^ stock	Enacyloxin, pyrrolnitrin, burkholdines, AFC-BC11, hydroxyquinolines	Mullins et al. (2019)
BCC0250	CEP0958, R-9927	CF patient sputum, Australia	Enacyloxin, pyrrolnitrin, burkholdines, AFC-BC11, hydroxyquinolines	Coenye et al. (2001); Mullins et al. (2019)
BCC0480	HI2427	Soil, USA	Enacyloxin, pyrrolnitrin, burkholdines, AFC-BC11, hydroxyquinolines	Mullins et al. (2019)
BCC1248	KW0-1	Maize rhizosphere, USA	Enacyloxin, pyrrolnitrin, burkholdines, AFC-BC11, phenazine	Ramette and Tiedje (2007); Mullins et al. (2019)
*Burkholderia gladioli*				
BCC0238	MA4	CF patient sputum, USA	Toxoflavin, gladiolin, caryoynencin, icosalides	Song et al. (2017); Jenner et al. (2019); Jones et al. (2020)
BCC0771	LMG 2216^T^, ATCC 10248^T^, DSM 4285^T^	*Gladiolus* sp. bulb, USA	Toxoflavin, gladiolin, caryoynencin, icosalides	Coenye et al. (1999); Jones et al. (2020)
BCC1622	AU17110	CF patient sputum, USA	Toxoflavin, gladiolin, caryoynencin, icosalides	Jones et al. (2020)
BCC1647	LMG 6882	*Gladiolus* sp. bulb, USA	Toxoflavin, gladiolin, caryoynencin, icosalides	Coenye et al. (1999); Jones et al. (2020)
BCC1665	AU19515	CF patient sputum, USA	Toxoflavin, enacyloxin, caryoynencin, icosalides, bongkrekic acid	Jones et al. (2020)
BCC1686	AU16339	CF patient sputum, USA	Toxoflavin, enacyloxin, caryoynencin, icosalides, bongkrekic acid	Jones et al. (2020)
BCC1678	AU14817	CF patient sputum, USA	Toxoflavin, enacyloxin, icosalides, bongkrekic acid, sinapigladioside^a^	Jones et al. (2020)
BCC1697	AU18435	CF patient sputum, USA	Toxoflavin, icosalides, bongkrekic acid	Jones et al. (2020)
BCC1701	AU19655	CF patient sputum, USA	Toxoflavin, enacyloxin, caryoynencin, icosalides, bongkrekic acid	Jones et al. (2020)
BCC1721	AU22444	CF patient sputum, USA	Toxoflavin, gladiolin, caryoynencin, icosalides	Jones et al. (2020)
BCC1806	AU14276	CF patient sputum, USA	Toxoflavin, gladiolin, caryoynencin, icosalides	Jones et al. (2020)
BCC1811	AU22765	CF patient sputum, USA	Toxoflavin, gladiolin, caryoynencin, icosalides	Jones et al. (2020)

a No biosynthetic gene cluster for sinapigladioside has been identified (Flórez et al. 2018) but the compound has been identified by HPLC detection (Jones et al. 2020).

### 2.2 Rapid screening method for the detection of specialised metabolites

**Fig. 1:**
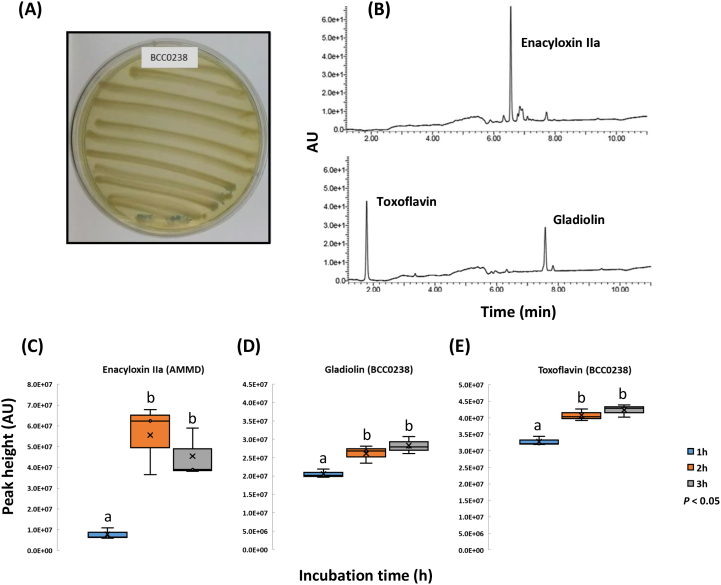
The detection of *Burkholderia* metabolites by HPLC and optimisation of solvent extraction time. (A) An example of bacterial growth (*B. gladioli* BCC0238) streaked on a 9.0 cm diameter BSMG agar plate for metabolite extraction grown at 30 °C for 72 h. (B) HPLC profiles of enacyloxin IIa produced by *B. ambifaria* AMMD (top panel) and gladiolin and toxoflavin produced by *B. gladioli* BCC0238 (bottom panel). (C) Increase in enacyloxin IIa extraction with time using dichloromethane from *B. ambifaria* AMMD. (D, E) Increase in gladiolin and toxoflavin extraction with time using dichloromethane from *B. gladioli* BCC0238. Means followed by the same letter are not significantly different according to the least significant difference test at *p* < 0.05 (*n* = 3): (C) LSD = 9.70E+06 AU, (D) LSD = 1.67E+06 AU, (E) LSD = 1.38E+06 AU. AU = absorbance units measured at 210–400 nm.

For specialised metabolite induction, bacterial inoculum was streaked (from a fresh overnight liquid culture; Fig. 1A) using a sterile swab (Fisher Scientific UK Ltd.) onto solidified (purified agar; Oxoid) basal salts medium (Hareland et al. 1975) consisting of (g l^−1^) K_2_HPO_4_.3H_2_O (4.25), NaH_2_PO_4_.H_2_O, (1.0), NH_4_Cl (2.0), MgSO_4_.7H_2_O (0.2), FeSO_4_.7H_2_O (0.012), MnSO_4_.H_2_O (0.003), ZnSO_4_.7H_2_O (0.003), CoSO_4_.7H_2_O (0.001), nitrilotriacetic acid trisodium salt (0.1), casamino acid (0.5), yeast extract (0.5) and supplemented with 4 g l^−1^ glycerol (BSMG; Mahenthiralingam et al. 2011). To ensure reproducibility, all BSMG plates contained 20 ml media and each plate was streaked 10 times (Fig. 1A) with one swab of bacteria. After incubation at 30 °C for 72 h, the microbial biomass was removed from the agar plate using a sterile cell scraper (Fisher Scientific UK Ltd) and a 20 mm agar disc cut from the metabolite-induced plate and then placed into a 30-ml wide-mouth amber glass bottle (to reduce exposure to light) with 0.5 ml dichloromethane (see Fig. S1). Acetonitrile and ethylacetate were also evaluated as solvents but were not used for follow up experiments as dichloromethane proved optimal (see Section 3). Metabolites were extracted by incubating for up to 3 h at room temperature (approximately 22 °C) on a rocking platform shaker (40 rpm). The solvent extract was carefully transferred from the bottle using a glass Pasteur pipette to avoid agar carry-over, centrifuged at 14,000 ×*g* and placed into 2.0 ml amber glass vials for reversed-phase HPLC analysis.

### 2.3 HPLC analysis

Extracts (20 μl injection volume) were analysed on a Waters® AutoPurification™ High Performance Liquid Chromatography (HPLC) System fitted with a reversed-phase analytical column (Waters® XSelect CSH C18, 4.6 × 100 mm, 5 μm) and a C18 SecurityGuard™ cartridge (Phenomenex) in series. Detection of compounds was by absorbance at 210–400 by a photo-diode array detector (PDA). Mobile phases consisted of (A) water with 0.1% formic acid and (B) acetonitrile with 0.1% formic acid with a flow rate of 1.5 ml min^−1^. Elution conditions were as follows: 0 to 1 min, 95% phase A/5% phase B; 1 to 9 min, gradient of phase A from 95 to 5% and gradient of phase B from 5% to 95%; 10 to 11 min, 5% phase A/95% phase B; 11 to 15 min, 95% phase A/5% phase B. Known specialised metabolites were identified by HPLC peak retention times and UV absorbance characteristics, and by referencing these to internal standards characterised by High Resolution Liquid Chromatography-Mass Spectrometry (LC-MS) and Nuclear Magnetic Resonance (NMR) as described (Mahenthiralingam et al. 2011; Song et al. 2017; Mullins et al. 2019; Jones et al. 2020).

Metabolite peak heights were calculated using MassLynx V4.1 software (www.waters.com) and differences in mean peak areas with treatment were determined by analysis of variance (ANOVA) with the least significant difference (LSD) test at *α* = 0.05 implemented in IBM SPSS Statistics v25. Additional statistics were done using a two-tailed *t*-test. Purified pyrrolnitrin (Sigma) was used as a standard to confirm HPLC detection and peak retention time of this specialised metabolite.

### 2.4 Metabolite induction and suppression assay with antibiotics

To investigate the use of trimethoprim as a gene expression elicitor of silent BGCs, the above rapid screening method for the detection of specialised metabolites was employed. BSMG agar was supplemented with low concentrations of trimethoprim (0, 0.5, 1.0, 2.0, 5.0, 10.0 μg ml^−1^), and inoculated with four strains of *Burkholderia gladioli* (BCC1665, BCC1678, BCC1686, BCC1701) in triplicate. All inoculated plates were incubated at 30 °C for 72 h and analysed by HPLC as described above.

For more rapid analysis, antimicrobial susceptibility testing (AST) discs (Oxoid) were placed into BSMG plates. Trimethoprim, rifampicin, chloramphenicol, minocycline, levofloxacin, tobramycin, ceftazidime, amikacin, and meropenem were examined as clinically relevant antibiotics (see Table 2 for the concentrations used). Essentially, molten BSMG agar was cooled to 50 °C and two AST discs were equally spaced in a 9 cm plastic Petri-dish prior to plate pouring and then adjusted so that they were beneath the agar using sterile forceps before the agar set. Antibiotic plates were streaked with two *B. ambifaria* (AMMD, BCC0191) and two *B. gladioli* strains (BCC0238, BCC1697) in duplicate, incubated at 30 °C for 72 h. A 20 mm disc was cut from the agar above the AST disc and placed into a 30-ml wide-mouth amber glass bottle with 0.5 ml dichloromethane and analysed for specialised metabolites as above.

**Table 2:** List of antimicrobial susceptibility testing (AST) discs used as metabolite inducers/suppressors in this study.

AST disc	Concentration (μg)	Disc abbreviation	Antibiotic class	Mechanism
Amikacin	30	AK30	Aminoglycoside	Protein synthesis inhibitor
Tobramycin	10	TOB10	Aminoglycoside	Protein synthesis inhibitor
Chloramphenicol	10	C10	Chloramphenicol	Protein synthesis inhibitor
Minocycline	30	MH30	Tetracycline	Protein synthesis inhibitor
Levofloxacin	1	LEV1	Fluoroquinolone	DNA synthesis inhibitor
Rifampicin	2	RD2	Ansamycin	RNA synthesis inhibitor
Ceftazidime	10	CAZ10	Cephalosporins	Cell wall synthesis inhibitor
Meropenem	10	MEM10	Carbapenem	Cell wall synthesis inhibitor
Trimethoprim	1.25	W1.25	DHFR inhibitor	Folic Acid synthesis inhibitor

## 3 Results and discussion

### 3.1 Optimization of the rapid screening method

Optimization of the rapid screening method was carried out using *Burkholderia* species identified as producers of the bioactive polyketides, enacyloxin IIa (Mahenthiralingam et al. 2011) and gladiolin (Song et al. 2017), from *Burkholderia ambifaria* strain AMMD and *B. gladioli* strain BCC0238, respectively. After growth of the bacteria for 72 h and removal of biomass, initial experiments evaluated the use of different volumes of extraction solvent (5, 2, 1, 0.5 ml) and injection volumes for HPLC analysis (2, 5, 10, 15, 20 μl); dichloromethane was used as the initial solvent to optimise the method, with acetonitrile and ethylacetate evaluated subsequently. It was observed that consistent and reproducible HPLC detection of enacyloxin IIa and gladiolin was obtained from 20 mm agar discs extracted with 0.5 ml dichloromethane and 20 μl sample injection volumes (Fig. 1B). After initial detection of compounds by HPLC and subsequent confirmation of peak identity by referencing to known standards confirmed by LC-MS (Mahenthiralingam et al. 2011; Song et al. 2017; Mullins et al. 2019; Jones et al. 2020), shorter solvent incubation times were investigated as a means to increase rapidity of the method with maximum extraction efficiency. Results showed that after 2 h incubation of the metabolite-induced agar disc in dichloromethane significantly higher (*n* = 3) levels of both enacyloxin IIa (*p* = 0.016) and gladiolin (*p* = 0.003) were detected than at 1 h, with no further increase after 3 h incubation (Fig. 1C). In addition, the azapteridine antibiotic, toxoflavin a known phytotoxin (Furuya et al. 1997; Lee et al. 2016) and antifungal (Li et al. 2019) compound produced by *B. gladioli* was also readily identified; toxoflavin significantly (*n* = 3, *p* = 0.001) increased in concentration with solvent incubation time up to 2 h (Fig. 1C). This extraction optimisation demonstrated that a range of known *Burkholderia* metabolites could be readily characterised using this rapid screening method. Acetonitrile and ethylacetate were also tested as extraction solvents, with HPLC analysis showing that all three metabolites (enacyloxin IIa, gladiolin and toxoflavin) could be easily detected in extracts, but at lower concentrations than with dichloromethane (data not shown). This clearly demonstrated that the rapid screening method can be easily modified for use with different solvents to allow extraction of other specialised metabolites dependent on their chemical characteristics and solubility in different solvents.

The use of reversed-phase HPLC in gradient mode is a technique widely used to evaluate compound diversity in organic solvent extracts of microbial specialised metabolites grown in liquid media (e.g. Higgs et al. 2001; Tormo et al. 2003; Rutledge and Challis 2015). However, its use directly from extracts from solid media is less frequent. The direct analysis of samples from standardised agar plates increased the high throughput nature of the protocol allowing for greater sample replication and reproducibility, and the investigation of multiple growth conditions. It also modelled biofilm and high-density surface growth conditions which are preferred by multiple bacteria and known to activate regulatory systems such as quorum sensing, essential for expression of certain antibiotics (e.g. enacyloxin IIa; Mahenthiralingam et al. 2011). In addition, the method also allows rapid screening and identification of new strains that naturally produce higher levels of desired compounds (see below). Downstream of HPLC, genetic engineering can aid compound identification by comparative metabolite analysis of gene knockout mutants and wild-type strains (Kunakom and Eustáquio 2019). For example, cepacin and its related HPLC peak was determined in *B. ambifaria* BCC0191 after the BGC encoding cepacin was disrupted through insertional mutagenesis (Mullins et al. 2019). Similarly, the use of known standard compounds analysed alongside metabolite extracts can also help identify unknown peaks. In the current study purified pyrrolnitrin was used to help identify this compound in extracts from *B. ambifaria* (see below). Ultimately, further analyses beyond HPLC such as mass identification by LC-MS or structure elucidation by NMR are required for accurate compound identification (Mahenthiralingam et al. 2011; Song et al. 2017). However, for initial metabolite profiling, optimisation of extraction conditions and identifying production strains, the protocol proved very useful.

### 3.2 Identification of suitable production strains of specialised metabolites

**Fig. 2:**
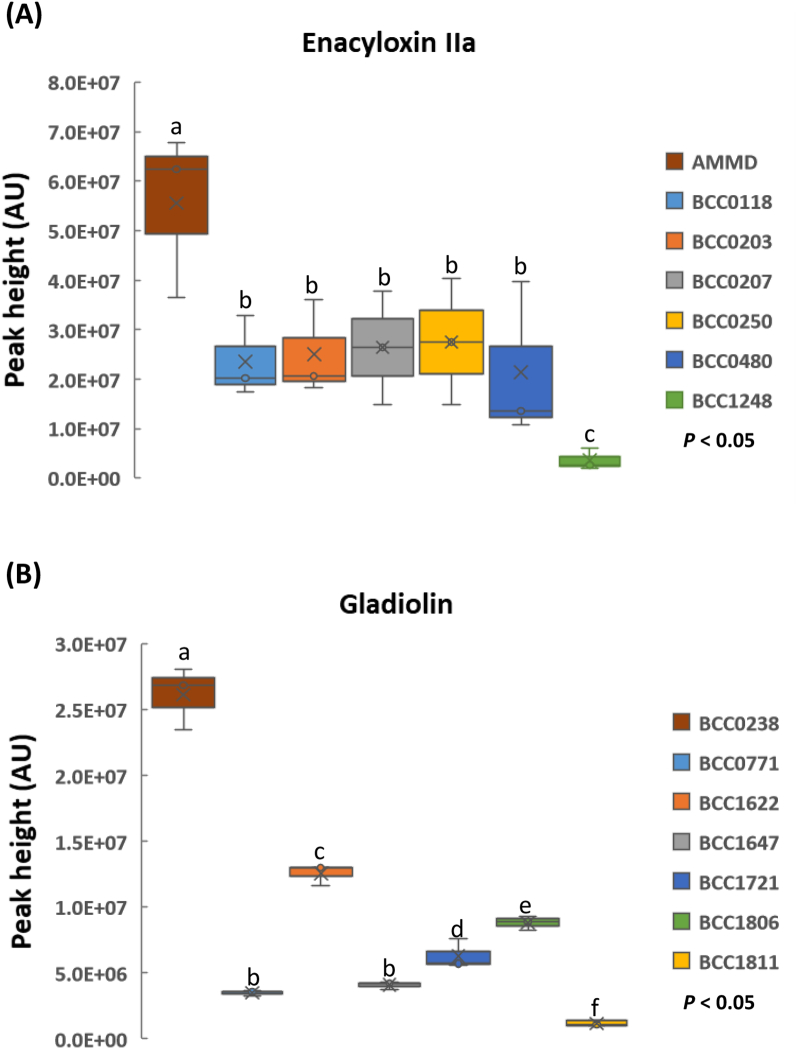
Screening and identification of high antibiotic production strains of *B. ambifaria* for enacyloxin IIa and *B. gladioli* for gladiolin. (A) enacyloxin IIa from *B. ambifaria* and (B) gladiolin from *B. gladioli* strains. All strains tested were grown on BSMG for 72 h at 30 °C. Means followed by the same letter are not significantly different according to the least significant difference test at *p* < 0.05 (*n* = 3): (A) LSD = 9.72E+06 AU, (B) LSD = 8.74E+05 AU. AU = absorbance units measured at 210–400 nm.

To identify high production strains for both enacyloxin IIa and gladiolin and facilitate large-scale purification of *Burkholderia* metabolites in sufficient quantities for future toxicity and efficacy testing, a panel of seven *B. ambifaria* and seven *B. gladioli* strains were screened using the rapid screening method. Results showed that strains *B. ambifaria* AMMD and *B. gladioli* BCC0238 were the optimum strains for the induction and production of enacyloxin IIa and gladiolin, respectively under the conditions tested. For both strains, significantly higher concentrations of antibiotics (*n* = 3; *p* < 0.01) were observed when compared with six other strains of the same species. Interestingly, the amounts of gladiolin produced by all *B. gladioli* strains evaluated were highly variable (Fig. 2), whereas enacyloxin IIa production was more consistent among the *B. ambifaria* strains tested, with the exceptions of AMMD (high concentration) and BCC1248 (low concentration). Two of the *B. ambifaria* isolates evaluated, BCC0207 and AMMD, were derived from the same original stock and are both representative of the *B. ambifaria* type strain AMMD. However, the strain designated AMMD in this study has been used routinely over a period of time to investigate enacyloxin IIa (Mahenthiralingam et al. 2011; Masschelein et al. 2019), and this may have inadvertently resulted in the selection of an improved strain with an altered genotype (Bunch and Harris 1986) for enacyloxin IIa production. Utilising strains that naturally produce high concentrations of specialised metabolites when available is preferential over engineering native hosts to improve metabolite production or heterologously expressing biosynthetic genes in other hosts, especially for recently identified, uncharacterised or large BGCs (Zhang et al. 2016). Natural efficient high metabolite producers are already equipped with the necessary cellular factors to produce the compound of interest, including those needed for precursor and product biosynthesis, pathway regulation, self-resistance and transport. *Burkholderia* strains shown to produce high concentrations of gladiolin and enacyloxin IIa identified during this study were subsequently used to enable the purification of sufficient antibiotic to investigate their activity on a panel of multi-drug resistant strains of urogenital pathogens, *Neisseria gonorrhoeae* and *Ureaplasma* spp. (Heath et al. 2020).

### 3.3 Effect of trimethoprim on specialised metabolites of *B. gladioli*

Previously, exposure to trimethoprim at subinhibitory concentrations has been reported as a global activator for *Burkholderia thailandensis* specialised metabolites, able to induce previously uncharacterised BGCs (Seyedsayamdost 2014; Okada et al. 2016; Li et al. 2020). To evaluate the trimethoprim induction phenomenon on different *Burkholderia* species, four strains of *B. gladioli* (BCC1665, BCC1678, BCC1686 and BCC1701) were grown on BSMG agar plates with a range of trimethoprim concentrations (0–10 μg ml^−1^) and metabolites were analysed as above. Initial experiments showed that the minimum inhibitory concentration (MIC) of trimethoprim for a number of strains of *B. gladioli* grown on BSMG agar plates (Table S1) or in TSB (Fig. S2) was between 2 and 10 μg ml^−1^.

**Fig. 3:**
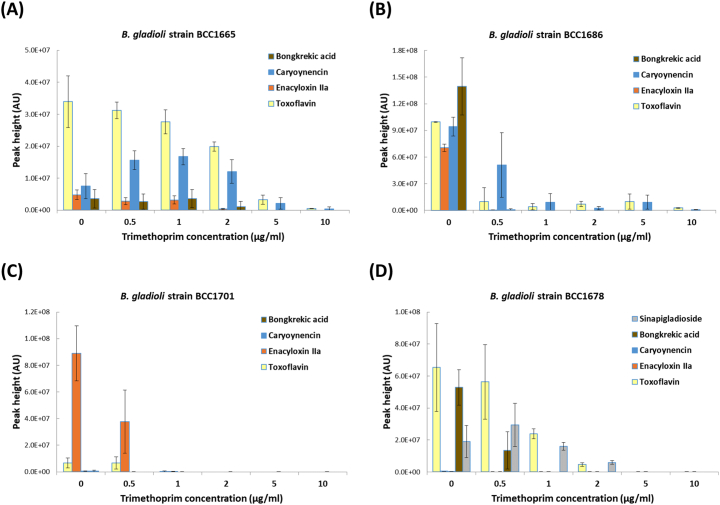
Effect of different concentrations of trimethoprim (0–10 μg ml^−1^) on the metabolite profile of different *Burkholderia gladioli* strains. (A) Strain BCC1665 (B) Strain BCC1686 (C) BCC1701 (D) BCC1678. Metabolites evaluated were toxoflavin, enacyloxin IIa, caryoynencin, bongkrekic acid and sinapigladioside (*n* = 9). The mean metabolite peak height (plus or minus the standard deviation of the mean) is plotted for each *B. gladioli* strain. AU = absorbance units measured at 210–400 nm.

In the presence of trimethoprim only known *B. gladioli* metabolites were detected by HPLC (toxoflavin, enacyloxin IIa, caryoynencin, bongkrekic acid and sinapigladioside) and quantified (Fig. 3), with no evidence of novel metabolites being detected. It was observed that instead of induction, trimethoprim was generally having a suppressive effect on the known *B. gladioli* metabolites, including the respiratory toxin bongkrekic acid (Anwar et al. 2017). All *B. gladioli* strains, except for strain BCC1665, showed a dramatic reduction in metabolite production at all trimethoprim concentrations analysed, including subinhibitory concentrations 0.5–1.0 μg ml^−1^ in a clear concentration-dependent manner (Fig. 3). Only *B. gladioli* BCC1665, showed some stimulation in the production of caryoynencin, but had similar levels of enacyloxin IIa and bongkrekic acid, and a decline in toxoflavin, when compared to the control without trimethoprim at concentrations between 0.5 and 2.0 μg ml^−1^. Closer examination of the data shows that three (BCC1678, BCC1686 and BCC1701) out of the four *B. gladioli* strains tested had a statistically significant reduction in bongkrekic acid production when exposed to subinhibitory concentrations of 1 μg ml^−1^ of trimethoprim (see Fig. S3, data from BCC1686 shown as an example).

Interestingly, the suppression of *Burkholderia* metabolites by the addition of subinhibitory concentrations of trimethoprim may have an unexpected benefit when used in a clinical setting. Cystic fibrosis (CF) patients often have polymicrobial infections of the lungs which can include members of the *Burkholderia cepacia* complex, strains of *B. gladioli*, and other bacteria (LiPuma 2010). For this reason, they are prescribed a cocktail of antibiotics including trimethoprim (Avgeri et al. 2009). The potential suppression of toxic metabolites like bongkrekic acid and toxoflavin by trimethoprim in CF patients with known *B. gladioli* infections would be clearly valuable. If toxins were produced by *B. gladioli* in the lung this would impose a further risk factor to CF patients. Previously, *B. gladioli* infections have been associated with severe symptoms caused by systemic infection including hypertrophic pulmonary osteoarthropathy (Jones et al. 2001) and death (Khan et al. 1996), although this has not been attributed to these toxic metabolites. Recently, the need to define *B. gladioli* strains which encode the bongkrekic acid gene cluster from strains that do not because of its link with food-poisoning (Jiao et al. 2013) has led to the reassessment of the species using phylogenomic approaches (Jones et al. 2020). All strains that were bongkrekic acid BGC positive, including CF patient isolates all clustered in one major group, and were referred to as *B. gladioli* Group 1 (Jones et al. 2020).

### 3.4 Effect of low concentrations of other antibiotics on *Burkholderia* specialised metabolites

Since trimethoprim was observed to have a clear suppressive effect on *B. gladioli* metabolite production and yet other antibiotics are known to stimulate natural product biosynthesis in other *Burkholderia* (Seyedsayamdost 2014), it was decided to test a range of different antibiotics on a panel of other *Burkholderia* species and screen their metabolite profiles using HPLC. To allow for more rapid screening, commercially available antimicrobial susceptibility testing (AST) discs impregnated with standardised concentrations of antibiotic were tested (Table 2). Preliminary experiments comparing the effect of trimethoprim within the agar (1.0 μg ml^−1^) against trimethoprim diffusing out from an AST disc (1.25 μg disc^−1^) was undertaken in order to determine the feasibility of the method (Fig. S3). Results showed that there was no significant difference between the effect of trimethoprim AST discs on bongkrekic acid production by *B. gladioli* BCC1686 when compared to a similar concentration of trimethoprim added directly to the growth media. Both treatments significantly (*n* = 4; *p* < 0.01) suppressed the metabolite, bongkrekic acid when compared to the control without trimethoprim.

Two *B. gladioli* (BCC0238, BCC1697) and two *B. ambifaria* (AMMD, BCC0191) strains were screened for changes in their specialised metabolites against a panel of 9 different antibiotics (covering 8 different antibiotic classes and 5 mechanisms of action; Table 2). Since the concentration of antibiotics used for the AST discs were determined by the manufacturer, analysis of the growth and inhibition of the bacteria was first assessed. All four strains of *Burkholderia* were inhibited by minocycline and meropenem, and both strains of *B. gladioli* additionally showed clear inhibition zones by the aminoglycosides, tobramycin and amikacin (Table S2 and Fig. S4). Both meropenem and minocycline are used to treat *Burkholderia* infections in addition to trimethoprim (Avgeri et al. 2009). MIC values reported for non-CF patient isolates of *Burkholderia cepacia* complex (Bcc) bacteria and clinical isolates of *B. gladioli* for both minocycline and meropenem are in the range 1–8 μg ml^−1^ (Zhou et al. 2007; Mazer et al. 2017), suggesting that the *B. ambifaria* and *B. gladioli* isolates used here would be inhibited by these antibiotics at the concentration employed. In addition, a study of clinical isolates of *B. gladioli* report that they are naturally susceptible to aminoglycosides (Segonds et al. 2009), whereas members of the Bcc (which includes *B. ambifaria*) are intrinsically resistant to this class of antibiotics (Nzula et al. 2002).

**Fig. 4:**
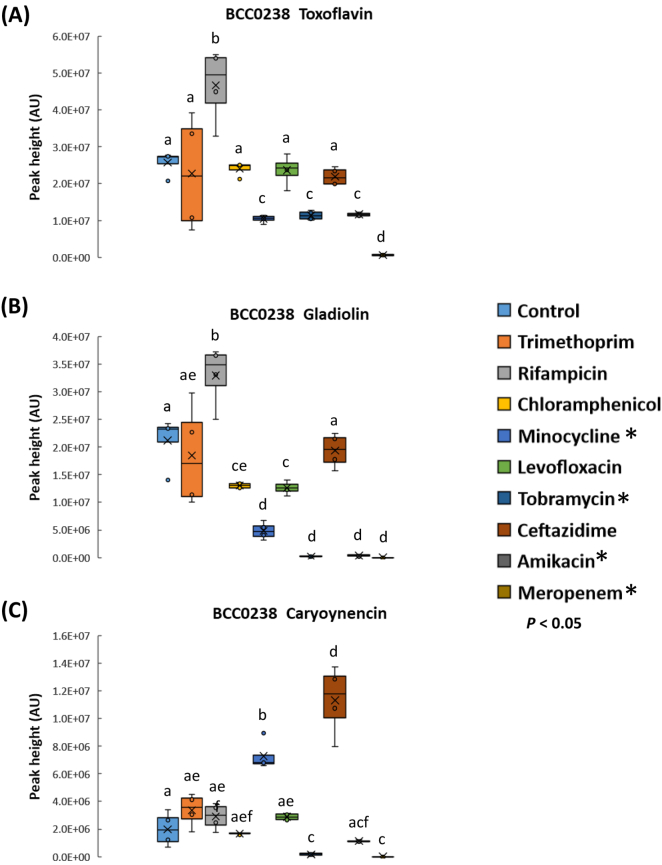
Effect of different antibiotics within AST discs on the metabolite production of *Burkholderia gladioli* BCC0238. Nine different antibiotics were screened as shown by the key on the right. The effect on the following metabolites was evaluated as shown in each panel: (A) toxoflavin (B) gladiolin and (C) caryoynencin production after 72 h at 30 °C. Antibiotic concentrations of AST discs are described in Table 2. Means followed by the same letter are not significantly different according to the least significant difference test at *p* < 0.05 (*n* = 4): (A) LSD = 4.46E+06 AU, (B) LSD = 2.80E+06 AU, (C) LSD = 7.70E+05 AU. Asterisks denote antibiotics that were inhibitory to BCC0238 growth. AU = absorbance units measured at 210–400 nm.

**Fig. 5:**
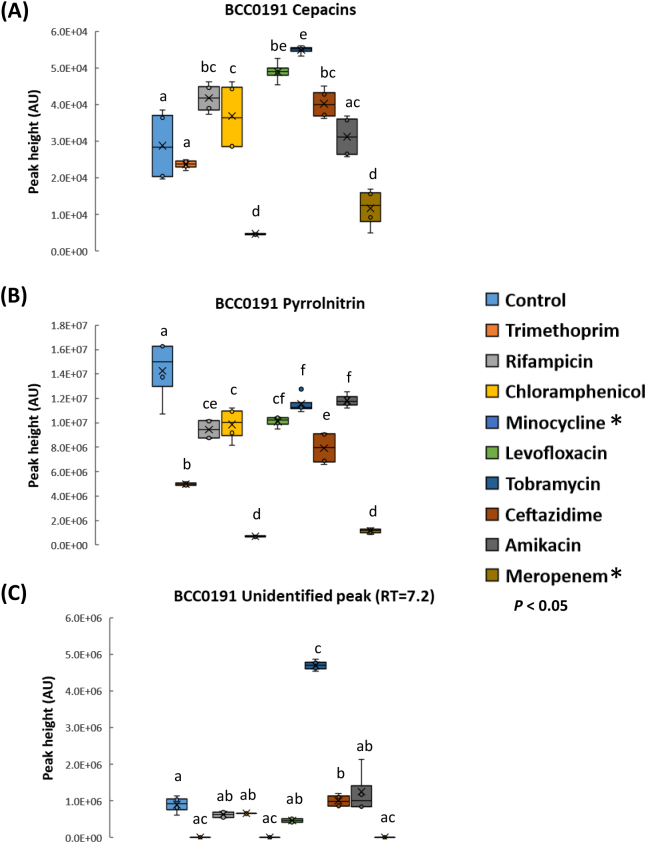
Effect of different antibiotics within AST discs on the metabolites of *Burkholderia ambifaria* BCC0191. Nine different antibiotics were screened as shown by the key on the right. The effect on the following metabolites was evaluated as shown in each panel: (A) cepacins (B) pyrrolnitrin and (C) and an unidentified metabolite peak (HPLC peak retention = 7.2 mins; UV absorbance = 301 nm) production after 72 h at 30 °C. Antibiotic concentrations of AST discs are described in Table 2. Means followed by the same letter are not significantly different according to the least significant difference test at *p* < 0.05 (*n* = 4): (A) LSD = 3.94E+03 AU, (B) LSD = 8.02E+05 AU, (C) LSD = 4.57E+05 AU. Asterisks denote antibiotics that were inhibitory to BCC0191 growth. AU = absorbance units measured at 210–400 nm, except cepacins measured at 240 nm.

However, despite the inhibition of growth by certain antibiotics, all nine antibiotics and four *Burkholderia* strain combinations were analysed by the rapid screening method. All the *Burkholderia* strains showed a differing response in terms of their metabolite profile to the panel of antibiotics tested (Fig. 4, Fig. 5; Figs. S5 and S6). The majority of strain-antibiotic treatment combinations resulted in a significant reduction in metabolite production or had no significant increase (Fig. 4, Fig. 5; Figs. S5 and S6). Certain interactions resulted in significant increases in known metabolite production or caused the induction of unidentified and potentially novel metabolites. For example, a significant increase (*n* = 4; *p* < 0.001) in both the phytotoxin, toxoflavin and the antibiotic, gladiolin were observed for *B. gladioli* BCC0238 in the presence of 2 μg rifampicin (Fig. 4). In an analogous fashion, the polyyne, caryoynencin produced by *B. gladioli* BCC0238 was significantly increased (*n* = 4; *p* < 0.001) in the presence of 10 μg ceftazidime.

A similar stimulatory effect of rifampicin was seen with *B. ambifaria* strain BCC0191 (Fig. 5) with an increase (*n* = 4; *p* = 0.003) in the production of the anti-oomycete polyyne compound, cepacin (Mullins et al. 2019). A significant increase (approx. 2-fold) in the stimulation of cepacin production above levels induced by the control and that by rifampicin was observed by 10 μg tobramycin (*n* = 4; *p* < 0.001). Interestingly, tobramycin also stimulated a 5-fold increase (*n* = 4; *p* < 0.001) in the production of an unidentified metabolite peak (HPLC peak retention = 7.2 mins; UV absorbance = 301 nm) by *B. ambifaria* BCC0191. Other significant increases in metabolites included that of: *B. ambifaria* AMMD antibiotic enacyloxin IIa by trimethoprim; an increase in pyrrolnitrin by chloramphenicol; induction of an unidentified metabolite peak (HPLC peak retention = 6.89 mins; UV absorbance = 330 nm) by chloramphenicol (Fig. S5). AntiSMASH analysis of the genomes from the two strains of *B. ambifaria* with novel metabolite peaks detected revealed them to have several uncharacterised BGCs including nonribosomal peptide synthetase (NRPS), polyketide synthase PKS and NRPS-type 1 PKS hybrid gene clusters (Mullins et al. 2019). Further investigation is needed to identify if the expression of any of these BGCs is activated by the presence of stimulatory antibiotics and if the novel metabolite peaks correspond to the specialised metabolite biosynthesis they encode.

Subinhibitory concentrations of antibiotics have long been known to have multiple effects on bacterial cells (Davies et al. 2006), but it is only recently with the advent of genome transcription analyses that these activities can been studied in detail. Low doses of rifampicin and erythromycin have been shown to change the expression of up to 5% of the transcripts in *Salmonella enterica*, with many of them being upregulated (Goh et al. 2002). Similarly, the addition of subinhibitory concentrations of trimethoprim to *B. thailandensis* resulted in both transcriptional and translational alterations, with 8.5% of the transcriptome and 5% of the proteome up or downregulated by more than 4-fold (Li et al. 2020). It was proposed that the low concentrations of trimethoprim inhibit one‑carbon metabolic processes, which leads to an accumulation of homoserine, that subsequently induces silent BGCs by a LuxR-type transcriptional regulator (Li et al. 2020). Understanding the mechanisms of antibiotic-based induction and/or suppression of *B. gladioli* and *B. ambifaria* metabolites seen in the current study would be interesting to address by global transcriptomic analysis. However, in the interim subinhibitory concentrations of antibiotics can clearly be used to discover specialised metabolites, improve the levels of specialised metabolites for further investigation, and to understand clinically if the presence of certain antibiotics drive detrimental toxin production in *Burkholderia*.

## 4 Summary

Here we have reported the use of a relatively simple, cost effective screening procedure for the investigation and optimisation of bacterial specialised metabolites. In this study we have been able to readily screen multiple strains of *B. gladioli* and *B. ambifaria* using a range of growth conditions and evaluating different elicitor molecules. A screening method that can provide rapid and reproducible profiles of specialised metabolites from *Burkholderia* species and other bacteria is a useful tool that can be utilised in research-based discovery of new antibiotics and other biotechnologically relevant metabolites. The method can be readily modified to investigate different induction conditions including, temperature, incubation time, media pH, carbon source and alternative metabolite inducers. Further understanding of how novel inducers or suppressors, such as low concentrations of antibiotics, act on bacterial specialised metabolite production has both medical and agricultural implications. Reducing expression of toxins from *Burkholderia* would benefit people with *Burkholderia* respiratory infections, such as those with cystic fibrosis, while activating the production of antimicrobial metabolites has important implications for natural product discovery and use of biopesticides in agriculture.

## Declaration of Competing Interest

The authors declare that they have no known competing financial interests or personal relationships that could have appeared to influence the work reported in this paper.

## Appendix A Supplementary data

Supplementary material Image 1
