# Supplementary material for: A rapid screening method for the detection of specialised metabolites from bacteria: Induction and suppression of metabolites from Burkholderia species
Source: J Microbiol Methods. 2020 Nov;178:106057. doi: 10.1016/j.mimet.2020.106057 (PMC7684528; doi:10.1016/j.mimet.2020.106057)
Supplement: Supplementary file 1 — Supplementary material [file mmc1.pdf]

Supplementary data

A rapid screening method for the detection of specialised metabolites from bacteria: induction and suppression of metabolites from *Burkholderia* species

Gordon Webster[1]*, Cerith Jones[1,2], Alex J. Mullins[1], Eshwar Mahenthiralingam[1]*

[1] Microbiomes, Microbes and Informatics Group, Organisms and Environment Division, School of Biosciences, Cardiff University, The Sir Martin Evans Building, Museum Avenue, Cardiff, Wales, CF10 3AX, UK. [2] Present address: School of Applied Sciences, Faculty of Computing, Engineering and Science, University of South Wales, Pontypridd, Wales, UK.

* Corresponding authors:

Gordon Webster, School of Biosciences, Cardiff University, The Sir Martin Evans Building, Museum Avenue, Cardiff, Wales, CF10 3AX, UK.

Tel: +44 (0)29 2087 5175, E-mail: websterg@cardiff.ac.uk

Eshwar Mahenthiralingam, School of Biosciences, Cardiff University The Sir Martin Evans Building, Museum Avenue, Cardiff, Wales, CF10 3AX, UK.

Tel: +44 (0)29 2087 5875, Fax: +44 (0)29 2087 4305, E-mail: mahenthiralingame@cardiff.ac.uk

E-mail addresses: websterg@cardiff.ac.uk (G. Webster), cerith.jones@southwales.ac.uk (C. Jones), mullinsa@cardiff.ac.uk (A.J. Mullins), mahenthiralingame@cardiff.ac.uk (E. Mahenthiralingam).

ORCID ID: 0000-0002-9530-7835 (G. Webster), 0000-0001-6275-0235, (C. Jones), 0000-0001-5804-9008 (A.J. Mullins), 0000-0001-9014-3790 (E. Mahenthiralingam).

Supplementary Figure S1 Webster et al.

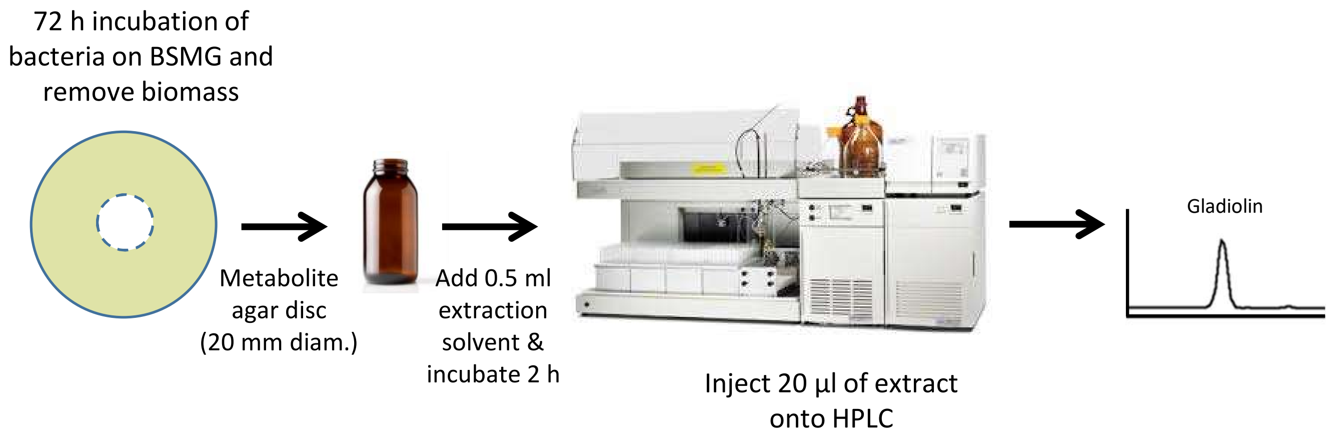

Supplementary Figure S1. Summary of the rapid screening method used for analysis of *Burkholderia* metabolites. BSMG = basal salts medium supplemented with glycerol. HPLC image amended from https://www.waters.com/waters/en_GB/AutoPurification-System/nav.htm?cid=10007147&locale=en_GB

Supplementary Figure S2 **Webster et al.**

(A)

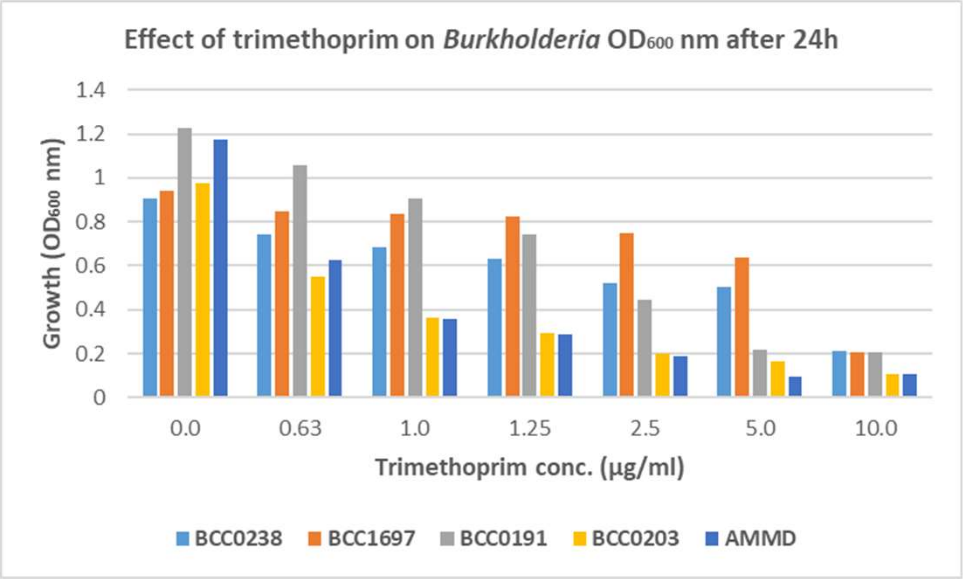

(B)

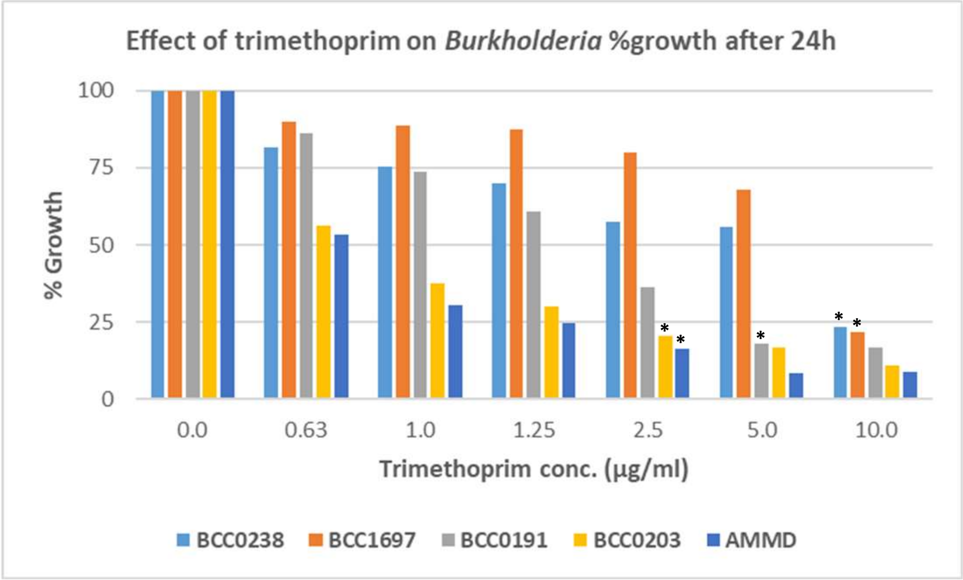

Supplementary Figure S2. Evaluating the minimum inhibitory concentration (MIC) ranges for trimethoprim on *B. gladioli* BCC0238, *B. gladioli* BCC1697, *B. ambifaria* BCC0191, *B. ambifaria* BCC0203 and *B. ambifaria* AMMD. A concentration range 0 to 10 µg ml^{-1} trimethoprim was tested. All strains were grown in TSB in a 96 well plate format for 24 hours at 30°C (*n* = 3) as described in Mullins *et al*. (2019). The colour coded key shows the identity of each strain screened. (A) shows the growth ($OD_{600\ nm}$) of each strain and (B) shows the % inhibition of growth compared to the control. The MIC was designated as the lowest trimethoprim concentration that reduced growth by greater than 75% compared to the control. The MIC for each strain is indicated by an asterisk.

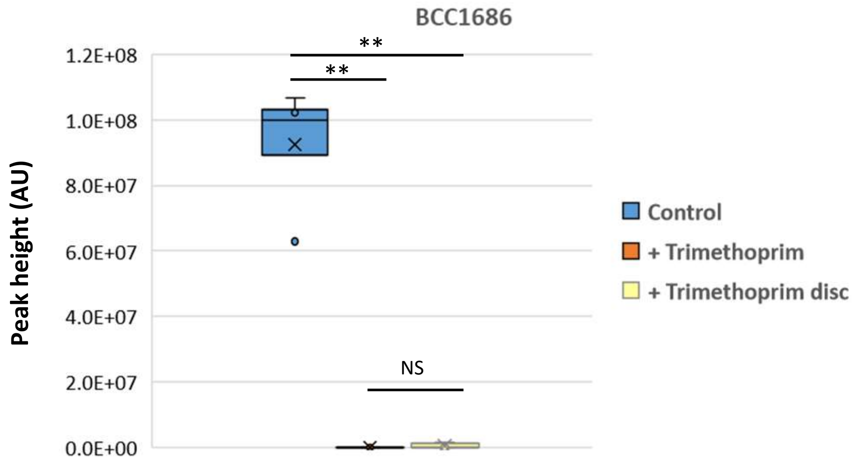

**$p < 0.01$: two-tailed t-test

Supplementary Figure S3. Subinhibitory concentrations of trimethoprim suppress bongkrekic acid (BA) production by *Burkholderia gladioli* BCC1686. Comparison between BA concentrations produced by *B. gladioli* grown on BSMG only with *B. gladioli* grown on BSMG with 1 µg ml^{-1} trimethoprim or BSMG with 1.25 µg trimethoprim AST discs for 72 h at 30°C (n = 4). Statistically significant metabolite peak heights are shown as indicated by the key. NS = not significant; AU = absorbance units measured at 210-400 nm.

Supplementary Figure S4 Webster et al.

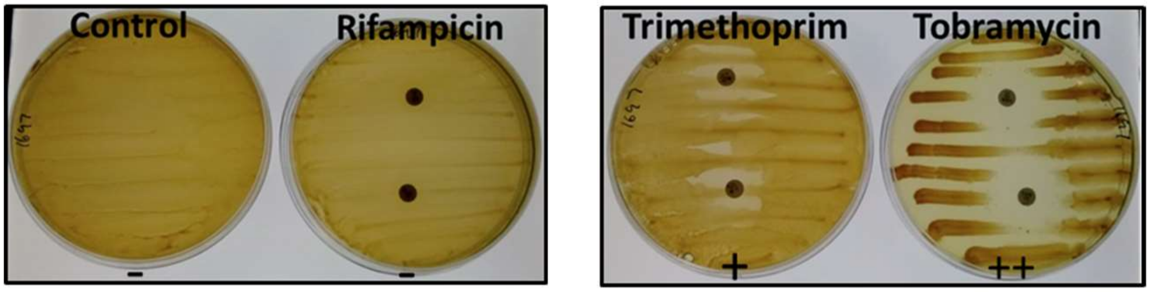

Supplementary Figure S4. Example of *Burkholderia* growth on BSMG agar plates supplemented with different AST discs. Growth of *B. gladioli* BCC1697 on BSMG, BSMG-rifampicin, BSMG-trimethoprim, and BSMG-tobramycin for 3 days at 30°C (see supplementary Table S2).

Supplementary Figure S5   Webster et al.

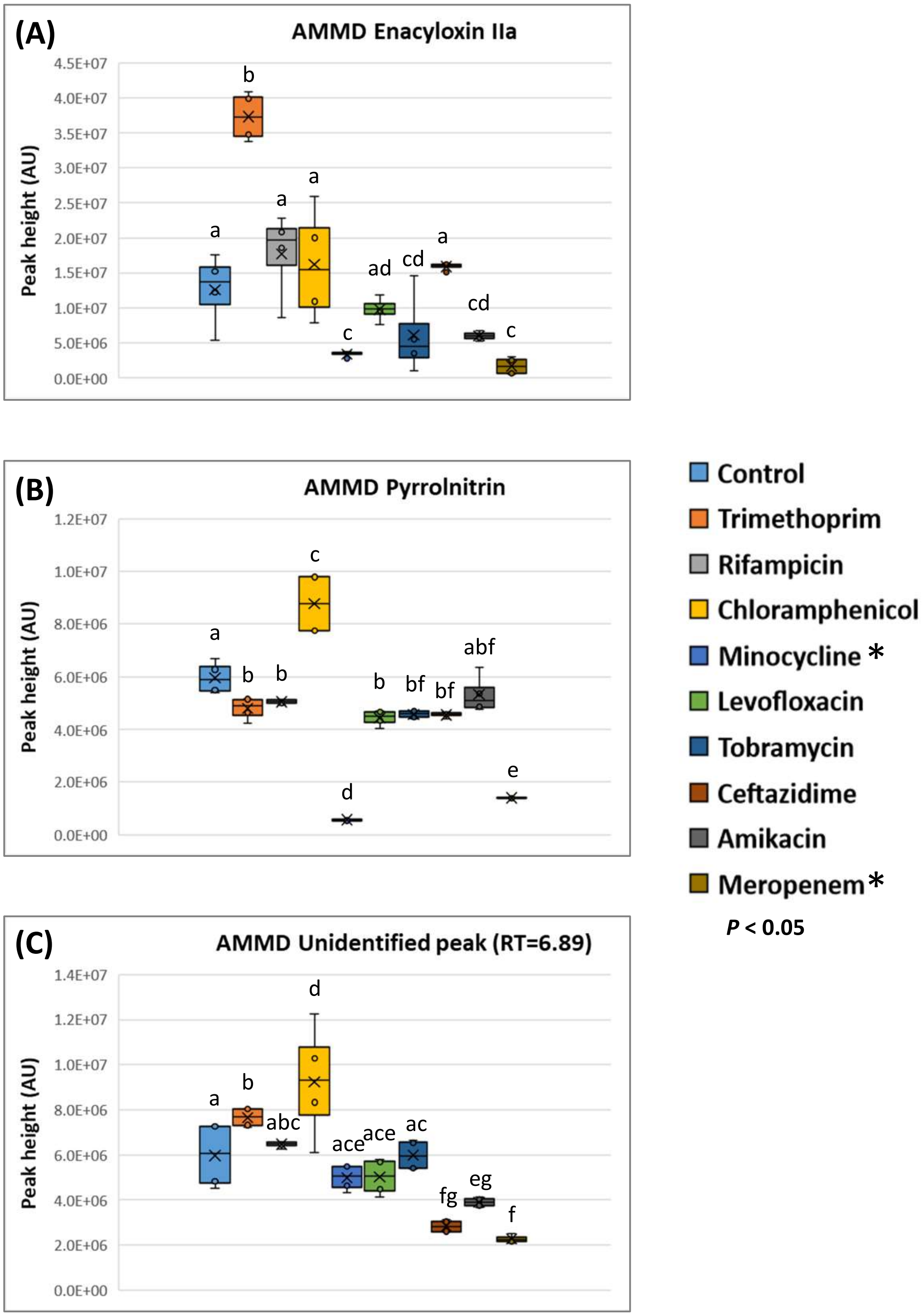

Supplementary Figure S5. Effect of different antibiotics within AST discs on the metabolites of *Burkholderia ambifaria* AMMD. Nine different antibiotics were screened as shown by the key on the right. The effect on the following metabolites was evaluated as shown in each panel: (A) enacyloxin IIa (B) pyrrolnitrin (C) and an unidentified metabolite peak (HPLC peak retention = 6.89 mins; UV absorbance = 330 nm) production after 72 hours at 30°C. Antibiotic concentrations of AST discs are described in Table 2. Means followed by the same letter are not significantly different according to the least significant difference test at p < 0.05 (n = 4): (A) LSD = 3.07E+06 AU, (B) LSD = 3.62E+05 AU, (C) LSD = 7.44E+05 AU. Asterisks denote antibiotics that were inhibitory to AMMD growth. AU = absorbance units measured at 210-400 nm.

Supplementary Figure S6 Webster et al.

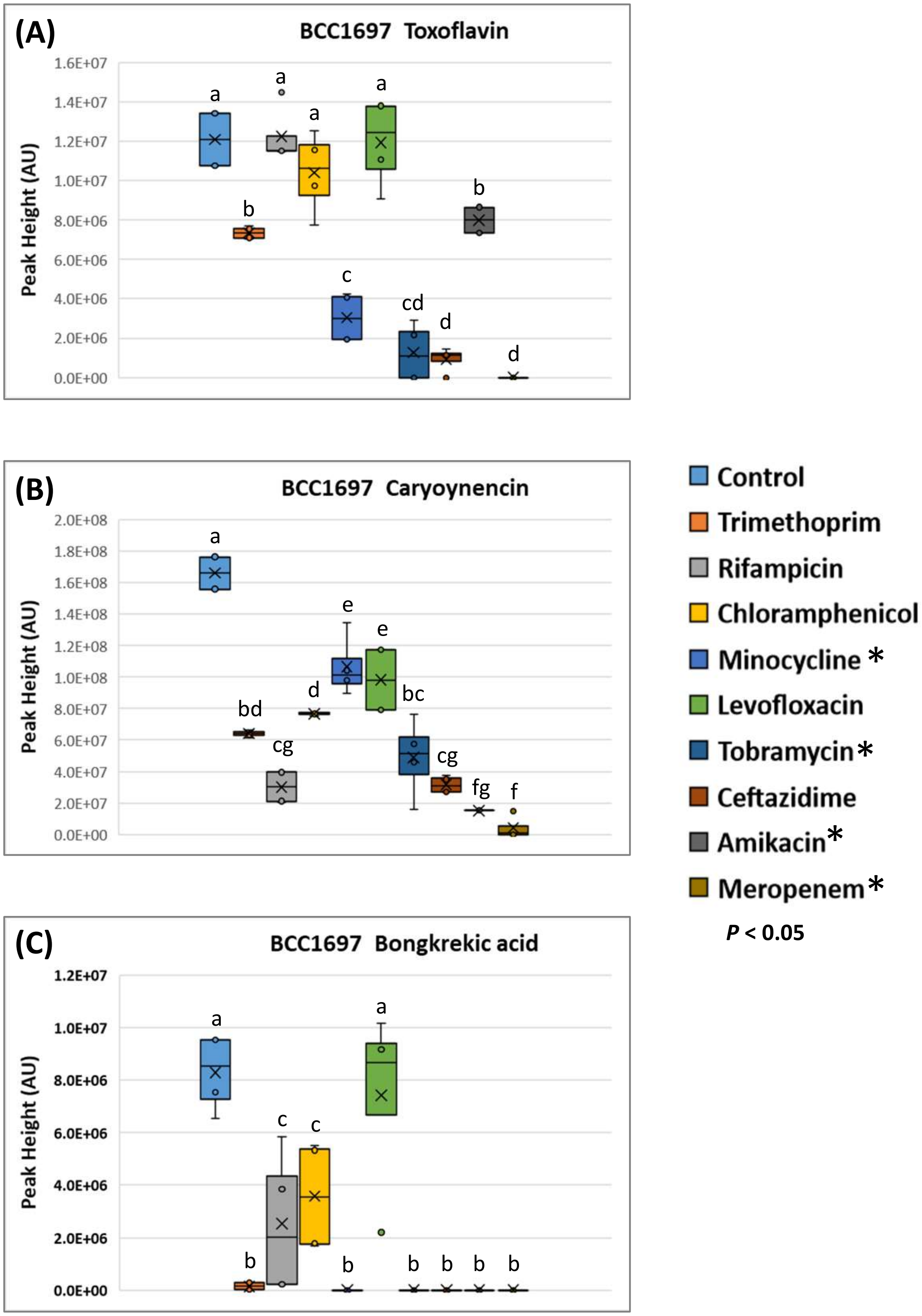

Supplementary Figure S6. Effect of different antibiotics within AST discs on the metabolites of *Burkholderia gladioli* BCC1697. Nine different antibiotics were screened as shown by the key on the right. The effect on the following metabolites was evaluated as shown in each panel: (A) toxoflavin (B) caryoynencin and (C) and bongkrekic acid production after 72 hours at 30°C. Antibiotic concentrations of AST discs are described in Table 2. Means followed by the same letter are not significantly different according to the least significant difference test at $p < 0.05$ (n = 4): (A) LSD = 9.82E+05 AU, (B) LSD = 9.63E+06 AU, (C) LSD = 1.17E+06 AU. Asterisks denote antibiotics that were inhibitory to BCC1697 growth. AU = absorbance units measured at 210-400 nm.

Supplementary Table S1. Evaluating the minimum inhibitory concentration (MIC) for trimethoprim on several *B. gladioli* strains grown on BSMG agar plates. A concentration range 0 to 64 µg ml^{-1} trimethoprim was tested. All strains were grown on BSMG for 24 hours at 30°C. The MIC was designated as the lowest trimethoprim concentration which showed no growth.

**Trimethoprim conc. (µg/ml)**	***B. gladioli* BCC0238**	***B. gladioli* BCC1665**	***B. gladioli* BCC1686**	***B. gladioli* BCC1697**
0	+	+	+	+
0.06	+	+	+	+
0.12	+	+	+	+
0.25	+	+	+	+
0.5	+	+	+	+
1	+	+	+	+
2	+	+	+	+
4	-	+/-	+/-	+/-
8	-	+/-	-	-
16	-	-	-	-
32	-	-	-	-
64	-	-	-	-

+ growth; +/- limited growth; - no growth

Supplementary Table S2. Growth and inhibition of *Burkholderia* species on BSMG agar supplemented with AST discs. Growth assessed after 72 hours incubation at 30°C.

Antibiotic disc	*B. gladioli* BCC0238	*B. gladioli* BCC1697	*B. ambifaria* BCC0191	*B. ambifaria* AMMD
Control	-	-	-	-
Trimethoprim	-	+	-	-
Rifampicin	-	-	-	-
Chloramphenicol	-	-	-	-
Minocycline	++	++	++	++
Levofloxacin	+	+	-	-
Tobramycin	++	++	-	-
Ceftazidime	-	-	-	-
Amikacin	++	++	-	-
Meropenem	++	++	++	++

++ clear zone of inhibition; + partial zone of inhibition; - no inhibition

See supplementary Figure S4 for an example.
